# The crucial role of *PpMYB10.1* in anthocyanin accumulation in peach and relationships between its allelic type and skin color phenotype

**DOI:** 10.1186/s12870-015-0664-5

**Published:** 2015-11-18

**Authors:** Pham Anh Tuan, Songling Bai, Hideaki Yaegaki, Takayuki Tamura, Seisuke Hihara, Takaya Moriguchi, Kenji Oda

**Affiliations:** NARO Institute of Fruit Tree Science, 2-1 Fujimoto, Tsukuba, Ibaraki 305-8605 Japan; Research Institute for Agriculture, Okayama Prefectural Technology Center for Agriculture, Forestry, and Fisheries, 1174-1 Koda-Oki, Akaiwa, Okayama 709-0801 Japan; Research Institute for Biological Sciences, Okayama Prefectural Technology Center for Agriculture Forestry, and Fisheries, 7549-1 Yoshikawa, Kibi-chou, Okayama 716-1241 Japan

**Keywords:** Anthocyanin, Japanese peach cultivars, MYB10 transcription factor, *Prunus persica*, Skin color, Transgenic tobacco

## Abstract

**Background:**

Red coloration of fruit skin is one of the most important traits in peach (*Prunus persica*), and it is mainly due to the accumulation of anthocyanins. Three *MYB10* genes, *PpMYB10.1*, *PpMYB10.2*, and *PpMYB10.3*, have been reported as important regulators of red coloration and anthocyanin biosynthesis in peach fruit. In this study, contribution of *PpMYB10.1*/*2*/*3* to anthocyanin accumulation in the fruit skin was investigated in the Japanese peach cultivars, white-skinned ‘Mochizuki’ and red-skinned ‘Akatsuki’. We then investigated the relationships between allelic type of *PpMYB10.1* and skin color phenotype in 23 Japanese peach cultivars for future establishment of DNA-marker.

**Results:**

During the fruit development of ‘Mochizuki’ and ‘Akatsuki’, anthocyanin accumulation was observed only in the skin of red ‘Akatsuki’ fruit in the late ripening stages concomitant with high mRNA levels of the last step gene leading to anthocyanin accumulation*, UDP-glucose:flavonoid-3-O-glucosyltransferase* (*UFGT*). This was also correlated with the expression level of *PpMYB10.1*. Unlike *PpMYB10.1*, expression levels of *PpMYB10.2*/*3* were low in the skin of both ‘Mochizuki’ and ‘Akatsuki’ throughout fruit development. Moreover, only *PpMYB10.1* revealed expression levels associated with total anthocyanin accumulation in the leaves and flowers of ‘Mochizuki’ and ‘Akatsuki’. Introduction of *PpMYB10.1* into tobacco increased the expression of tobacco *UFGT*, resulting in higher anthocyanin accumulation and deeper red transgenic tobacco flowers; however, overexpression of *PpMYB10.2*/*3* did not alter anthocyanin content and color of transgenic tobacco flowers when compared with wild-type flowers. Dual-luciferase assay showed that the co-infiltration of *PpMYB10.1* with *PpbHLH3* significantly increased the activity of *PpUFGT* promoter. We also found close relationships of two *PpMYB10.1* allelic types, MYB10.1-1/MYB10.1-2, with the intensity of red skin coloration.

**Conclusion:**

We showed that *PpMYB10.1* is a major regulator of anthocyanin accumulation in red-skinned peach and that it activates *PpUFGT* transcription. *PpMYB10.2*/*3* may be involved in functions other than anthocyanin accumulation in peach. The peach cultivars having two MYB10.1-2 types resulted in the white skin color. By contrast, those with two MYB10.1-1 or MYB10.1-1/MYB10.1-2 types showed respective red or pale red skin color. These findings contribute to clarifying the molecular mechanisms of anthocyanin accumulation and generating gene-based markers linked to skin color phenotypes.

**Electronic supplementary material:**

The online version of this article (doi:10.1186/s12870-015-0664-5) contains supplementary material, which is available to authorized users.

## Background

Peach (*Prunus persica*) is an important deciduous fruit, and its total production is ranked as 4th after grape, apple, and pear worldwide. China is the world’s leading producer of peach fruit, accounting for about 57 % of the total production. In Japan, peach is ranked 6^th^ in production, after mandarin, apple, pear, persimmon, and grape in 2012. Fruit skin color is one of the most important traits for the commercial value of peach fruit, and it is mainly determined by the content and composition of anthocyanins for red color or carotenoids for yellow color [1, 2]. With respect to carotenoid accumulation, yellow- and white-skinned types have been found, and the trait is controlled by a single *Y*/*y* locus in linkage group 1 [3, 4]. Recently, characterization of the *Y*/*y* locus has been reported by several research groups; *carotenoid cleavage deoxygenase 4* (*CCD4*) has been identified as a regulator of yellow pigmentation, and loss of function of *CCD4* results in the yellow-skinned type [5–9]. In contrast, red coloration of red-skinned peach depends on the accumulation of anthocyanins, which are water-soluble pigments of the flavonoid biosynthetic pathway. The intensity of red coloration is known to show variations depending on cultivars and strains, which suggests that red coloration is genetically controlled. Moreover, anthocyanin accumulation in the skin largely depends on environmental factors such as light and temperature conditions [10–12]. Most Japanese cultivars, including ‘Akatsuki’, show red skin color when environmental conditions are appropriate, while some Japanese cultivars, such as ‘Mochizuki’, seldom accumulate anthocyanin; therefore, this type of cultivar is suitable for canned processing. In Japan, red-skinned peach has a generally high market value, so farmers sometimes use the paper-bagging treatment for enhancing skin color, although production of white-skinned peach by using red-skinned cultivars (called “Hakuto”) has been established in Okayama Prefecture in Japan (http://world.momotaros.com/peach.html).

**Fig. 1:**
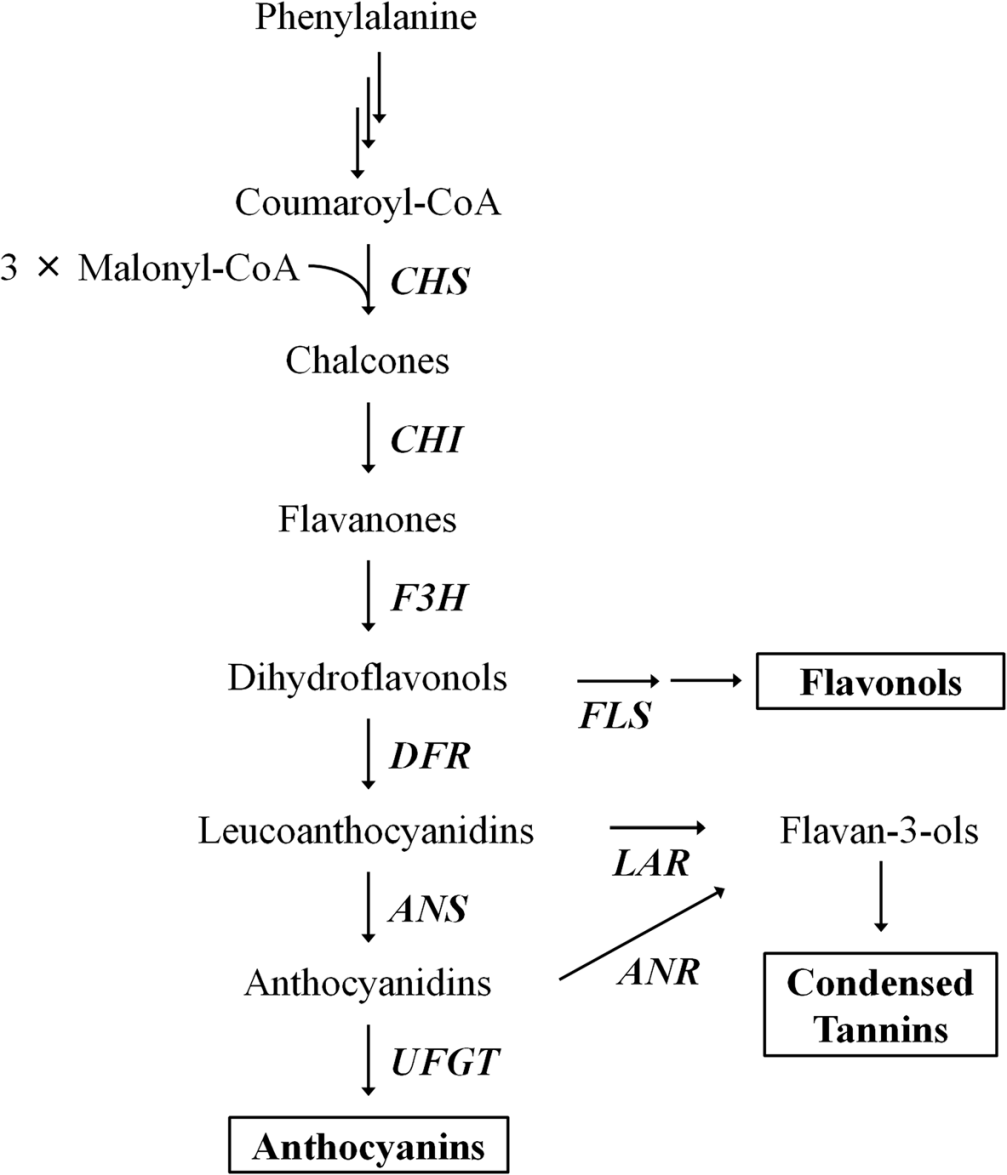
Flavonoid biosynthetic pathway in plants. CHS, Chalcone synthase; CHI, chalcone isomerase; F3H, flavanone 3-hydroxylase; DFR, dihydroflavonol-4-reductase; ANS, anthocyanidin synthase; UFGT, UDP-glucose:flavonoid-3-*O*-glucosyltransferase

The molecular mechanism underlying anthocyanin accumulation has been well-characterized in fruit trees [13–15]. Recently, many structural genes involved in the anthocyanin biosynthetic pathway and various transcription factors have been identified and characterized (Fig. 1). Of these, MYB transcription factor genes were often found to be the major determinant of anthocyanin accumulation by acting together with basic helix-loop-helix (bHLH) and WD40 proteins (termed the MBW complex) to activate key anthocyanin biosynthetic genes [15–17]. In grape, MYB genes contribute to anthocyanin biosynthesis via expression of *UFGT* [18, 19]. In apple, MYBs are involved in the activation of anthocyanin biosynthetic genes, and they regulate the accumulation of anthocyanin in fruit [20, 21]. In pear, the transcription level of MYB10 in the skin was positively correlated with anthocyanin biosynthetic gene pathway and anthocyanin biosynthesis [22, 23]. In peach, three *MYB10* genes, *PpMYB10.1* (Genome Database for Rosaceae accession number: ppa026640m), *PpMYB10.2* (ppa016711m), and *PpMYB10.3* (ppa020385m), localized in a genomic region of linkage group 3 where the *Anther color* (*Ag*) trait is located, have been reported as important regulators of anthocyanin biosynthesis in peach fruit [24]. *PpMYB10.2* positively regulates the promoter of *PpUFGT*, which is the only gene that shows a similar expression pattern to that of anthocyanin accumulation in peach skin during fruit development [25]. Rahim et al. [26] showed that the expression levels of *PpMYB10.1* and *PpMYB10.3* correlate with anthocyanin content as well as expression levels of key structural genes in the anthocyanin biosynthetic pathway. Our preliminary study on anthocyanin accumulation using red-skinned cultivars showed high expression levels of *PpMYB10.1* but quite low levels of *PpMYB10.3*, which may indicate that anthocyanin accumulation in peach skin is dominantly regulated by only *PpMYB10.1*.

The aim of this study was to evaluate the molecular characterization of the three *PpMYB10* genes by using Japanese peach cultivars. We first used two Japanese peach cultivars, white-skinned ‘Mochizuki’ and red-skinned ‘Akatsuki’, to study the relationship between the transcription levels of *PpMYB10.1*/*2*/*3*, anthocyanin biosynthetic genes, and anthocyanin accumulation in fruit skin during fruit development. Next, we analyzed overexpression of *PpMYB10.1*/*2*/*3* in tobacco and regulation of *PpUFGT* promoter activity by *PpMYB10.1*. Finally, we investigated the intensity of red coloration in the peach skin based on the allelic type of *PpMYB10.1*.

## Results

### Total anthocyanin content and expression analysis of anthocyanin biosynthetic genes in fruit skin of ‘Mochizuki’ and ‘Akatsuki’

**Fig. 2:**
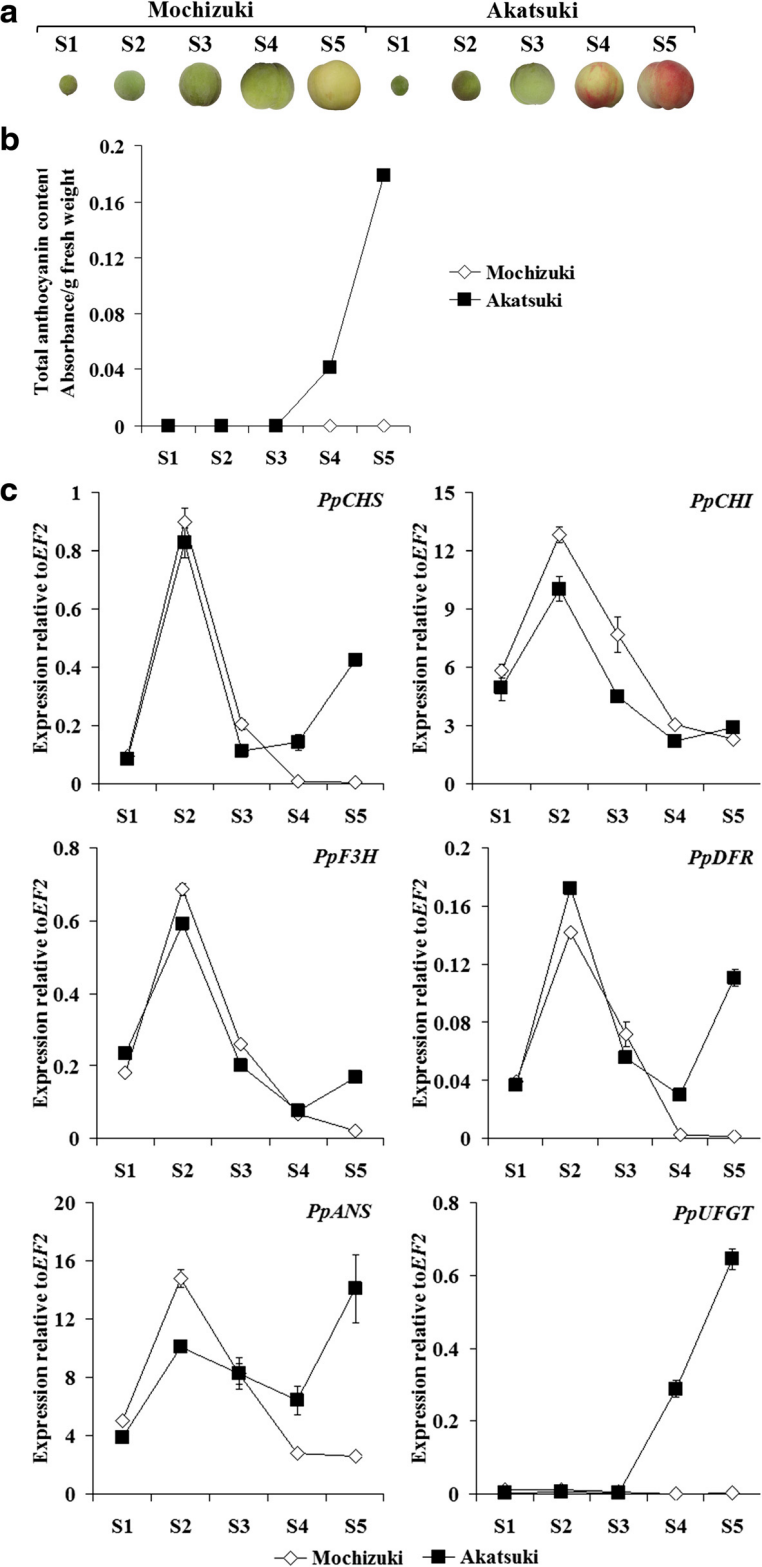
**a** Photographs of fruit skin. **b** Total anthocyanin content. **c** Expression levels of structural genes involved in the anthocyanin biosynthetic pathway in the skin of ‘Mochizuki’ and ‘Akatsuki’ during fruit development. Height of the *bars* and *error bars* shows the mean and standard error, respectively, from three independent measurements

The fruit skin of ‘Mochizuki’ is green in the first four stages and pale-yellow in the ripening stage, stage 5 (Fig. 2a). ‘Akatsuki’ fruit skin is also green in the early stages and partially or nearly red in stages 4 and 5, respectively (Fig. 2a). Total anthocyanin content in fruit skin was measured (Fig. 2b). As expected, white-skinned ‘Mochizuki’ did not show anthocyanin in the skin throughout fruit development. Anthocyanin was also not found at the beginning of ‘Akatsuki’ fruit development, and it only appeared in stage 4 and increased to a great extent in stage 5. This is in accordance with the red coloration observed in stages 4 and 5 of ‘Akatsuki’ skin.

Expression profiles of structural genes involved in anthocyanin biosynthesis were examined using quantitative real-time PCR (qRT-PCR) (Fig. 2c). In general, genes involved in the upstream pathway, including *PpCHS*, *PpCHI*, *PpF3H*, and *PpDFR*, showed similar expression patterns in the skin of ‘Mochizuki’ and ‘Akatsuki’ during fruit development; the expression levels increased from stage 1, peaked at stage 2, and then decreased in the last three stages. This is also the expression pattern of *PpANS* in the skin of ‘Mochizuki’, while *PpANS* revealed the highest expression level in stage 5 for ‘Akatsuki’. The mRNA level of *PpUFGT* was low in ‘Mochizuki’ skin in all stages, and it was also low in ‘Akatsuki’ skin in the three early stages and increased in stages 4 and 5. Although the expression levels of *PpCHS*, *PpDFR*, and *PpANS* were higher in the skin in stages 4 and 5 of ‘Akatsuki’ fruit, only the last step gene that directly leads to anthocyanin accumulation, *PpUFGT* showed an expression pattern tightly correlated with anthocyanin accumulation in the skin throughout fruit development in ‘Mochizuki’ and ‘Akatsuki’. These results suggest that *PpUFGT* is the key gene for anthocyanin accumulation in the skin of ‘Mochizuki’ and ‘Akatsuki’ fruit. Therefore, we characterized *MYB* genes that could act as a trans-factor of *PpUFGT*.

### Expression analysis of *PpMYB10.1*/*2*/*3* in the skin of ‘Mochizuki’ and ‘Akatsuki’ fruit

**Fig. 3:**
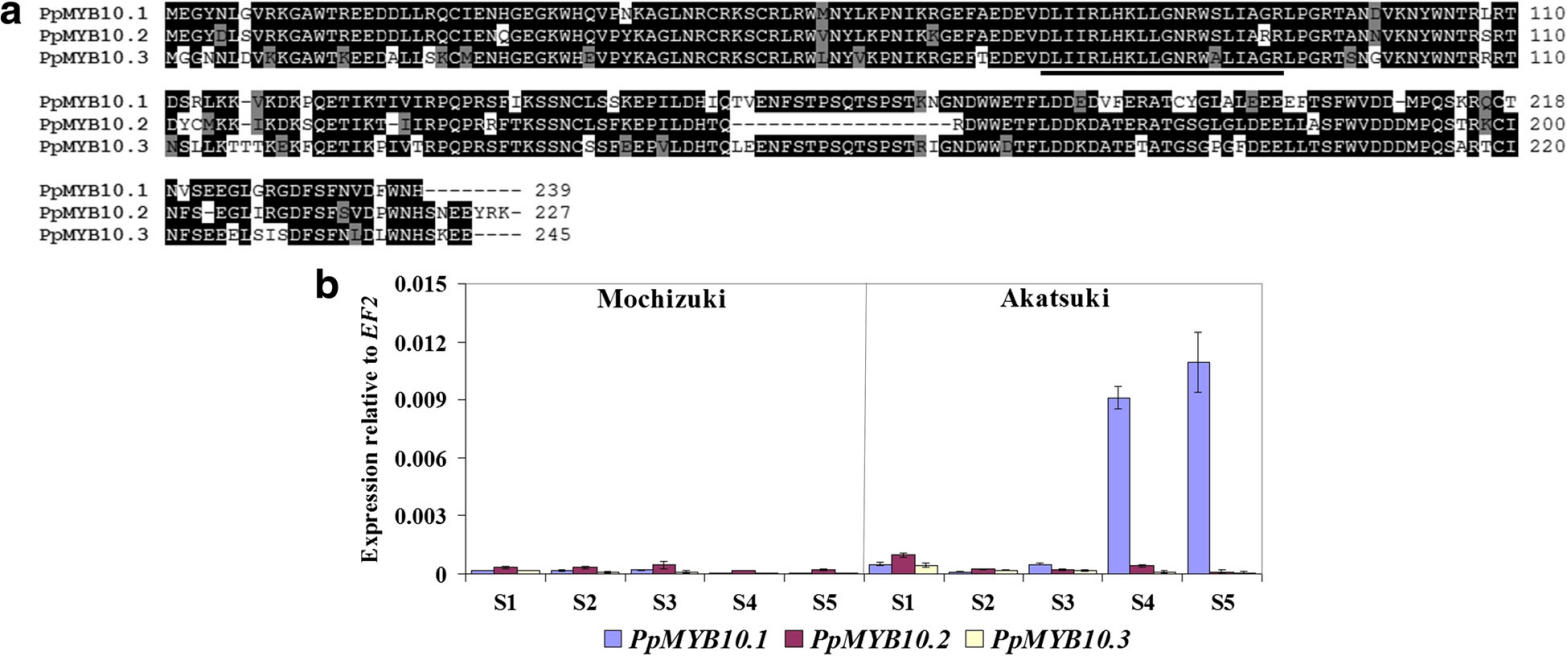
**a** Amino acid sequence alignment of PpMYB10.1/2/3. Sequences were retrieved from Genome Database for Rosaceae website (https://www.rosaceae.org/species/prunus/prunus_persica) in which red peach cultivar ‘Lovell’ was used to sequence. The solid underline is the R/B-like bHLH binding motif ([DE]Lx_2_[RK]x_3_Lx_6_Lx_3_R). **b** Expression levels of *PpMYB10.1*/*2*/*3* in the skin of ‘Mochizuki’ and ‘Akatsuki’ during fruit development. Height of the *bars* and *error bars* shows the mean and standard error, respectively, from three independent measurements

*PpMYB10.1*, *PpMYB10.2*, and *PpMYB10.3* are localized near each other in linkage group 3. Due to high similarity of the nucleotide sequences among these *PpMYB10s* (Fig. 3a), qRT-PCR primers for *PpMYB10.1*/*2*/*3* were manually designed on the basis of divergent nucleotide sequences between them. Real-time PCR products were then carefully tested for specificity by cloning into a pCR2.1-TOPO vector, and seven individual plasmid clones were sequenced to ensure product specificity for each primer set. Expression levels of *PpMYB10.1*/*2*/*3* were low in the skin during the five developmental stages of ‘Mochizuki’ fruit (Fig. 3b). In ‘Akatsuki’ skin, expression levels of *PpMYB10.1*/*2*/*3* were also low at the beginning of fruit development; then, transcription levels of *PpMYB10.1* dramatically increased in stages 4 and 5, while expression levels of *PpMYB10.2*/*3* remained low throughout fruit maturation. High mRNA levels of *PpMYB10.1* found in stages 4 and 5 were correlated with anthocyanin content and red pigmentation, which were observed only in these two ripening stages of ‘Akatsuki’ fruit. These results demonstrated that *PpMYB10.1* alone is responsible for anthocyanin accumulation in the skin of ‘Akatsuki’. To confirm this assumption, we then created transgenic tobacco plants that overexpressed the three *PpMYB10* genes.

### Characterization of transgenic tobacco plants that overexpressed *PpMYB10.1*/*2*/*3*

**Fig. 4:**
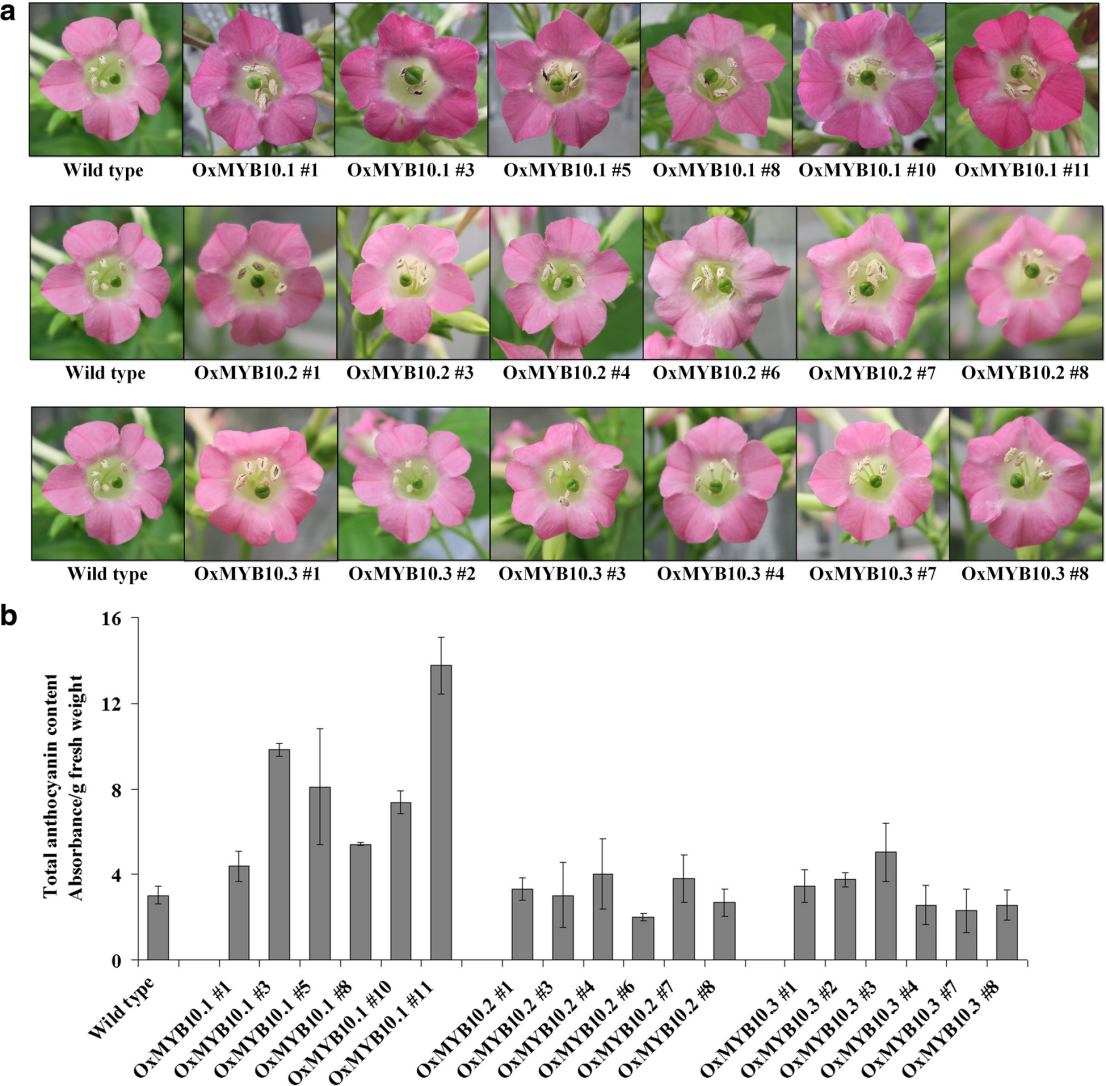
Photographs (**a**) and total anthocyanin content (**b**) of transgenic tobacco flowers overexpressing *PpMYB10.1*/*2*/*3*. Height of the *bars* and *error bars* shows the mean and standard error, respectively, from three independent measurements

**Fig. 5:**
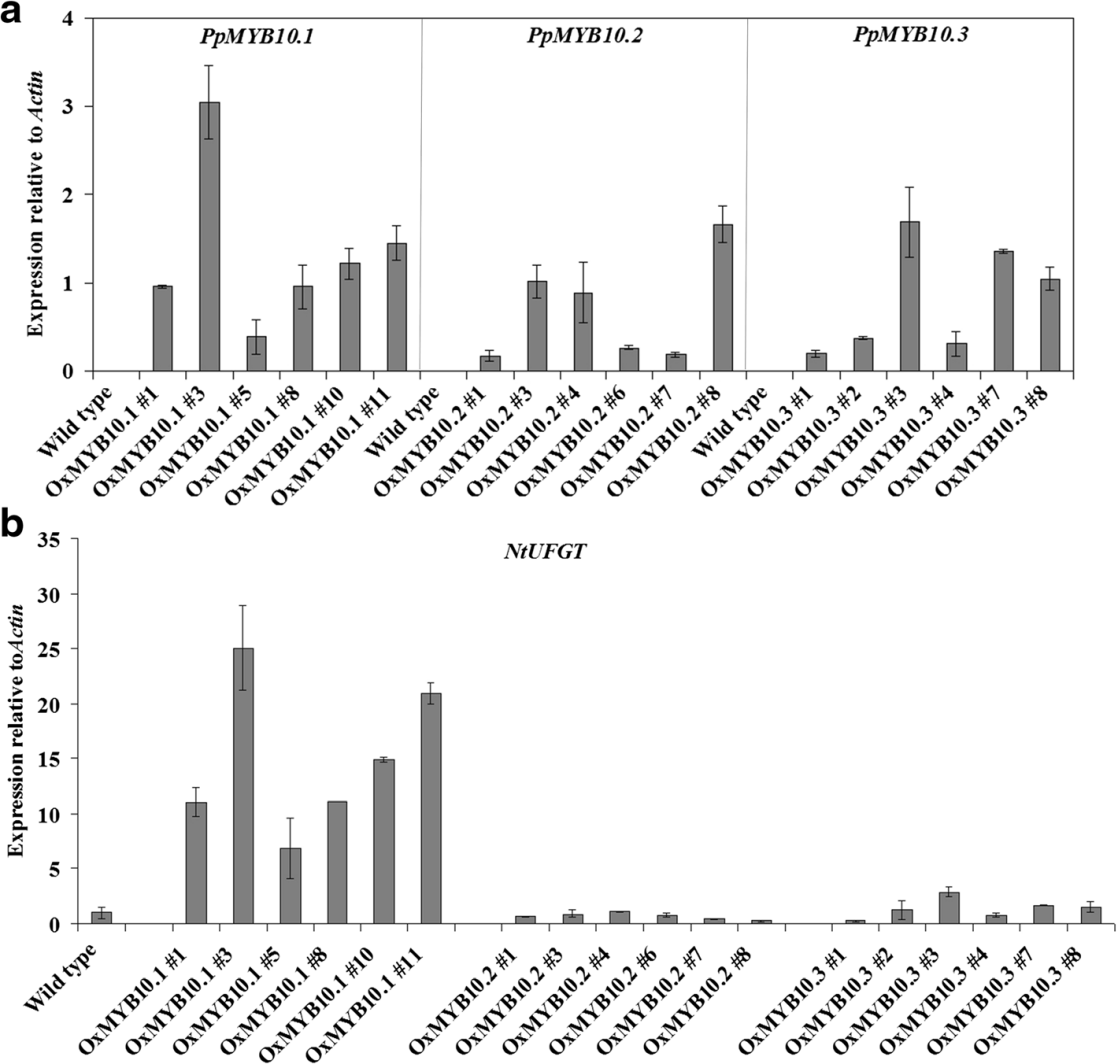
Expression levels of *PpMYB10.1*/*2*/*3* transgenes (**a**) and *NtUFGT* (**b**) in transgenic tobacco flowers. Height of the *bars* and *error bars* shows the mean and standard error, respectively, from three independent measurements

ORFs of *PpMYB10.1*/*2*/*3* driven by the CaMV 35S promoter were introduced into *Nicotiana tabacum* SR1 by using *Agrobacterium tumefaciens* strain LBA4404. Regenerated plants on plates containing 50 mg/L of kanamycin were examined for the presence of transgenes by using PCR with extracted genomic DNA (Additional file 1: Figure S1a). For each overexpression construct, six independent lines of transgenic plants showing the presence of the corresponding transgene were selected to transfer to the soil and grown under greenhouse conditions. As observed in Fig. 4a, introduction of *PpMYB10.1* resulted in a deeper red color in transgenic tobacco flowers when compared with the wild-type tobacco flowers. The capsule skin of transgenic tobacco plants overexpressing *PpMYB10.1* also displayed a pale red color, while the capsule skin of wild-type tobacco was green (Additional file 1: Figure S1b). Transgenic tobacco plants transformed with *PpMYB10.2*/*3* showed no coloration differences with respect to flowers when compared with wild-type flowers (Fig. 4a). This color observation reflected that obviously higher anthocyanin accumulation was only found in the flowers of six *PpMYB10.1* transgenic tobacco lines (Fig. 4b). To investigate the regulation of branching genes for specific flavonoid groups, such as flavonols and tannins, by transgenes, expression levels of transgenes *PpMYB10.1*/*2*/*3* and *N. tabacum FLS*, *LAR*, *ANR*, and *UFGT* were analyzed in transgenic tobacco flowers (Additional file 2: Figure S2). The results showed that all *PpMYB10.1*/*2*/*3* mRNAs were transcribed (Fig. 5a). Moreover, overexpression of *PpMYB10.1* substantially upregulated only *NtUFGT* expression, and overexpression of *PpMYB10.2*/*3* did not markedly alter the transcription of all four examined genes in transgenic tobacco flowers (Fig. 5b, Additional file 2: Figure S2). In addition, expression level of *NtUFGT* was consistent with the expression level of *PpMYB10.1* transgene in six independent transgenic lines (Fig. 5). Taken together, only *PpMYB10.1* can activate tobacco *NtUFGT*, resulting in higher anthocyanin accumulation and deeper red color in transgenic tobacco flowers. Then, what are the functions of *PpMYB10.2* and *PpMYB10.3*? To confirm this, we analyzed expressions in leaves and flowers of ‘Mochizuki’ and ‘Akatsuki’.

### Total anthocyanin content and expression analysis of *PpMYB10.1*/*2*/*3* in leaves and flowers of ‘Mochizuki’ and ‘Akatsuki’

**Fig. 6:**
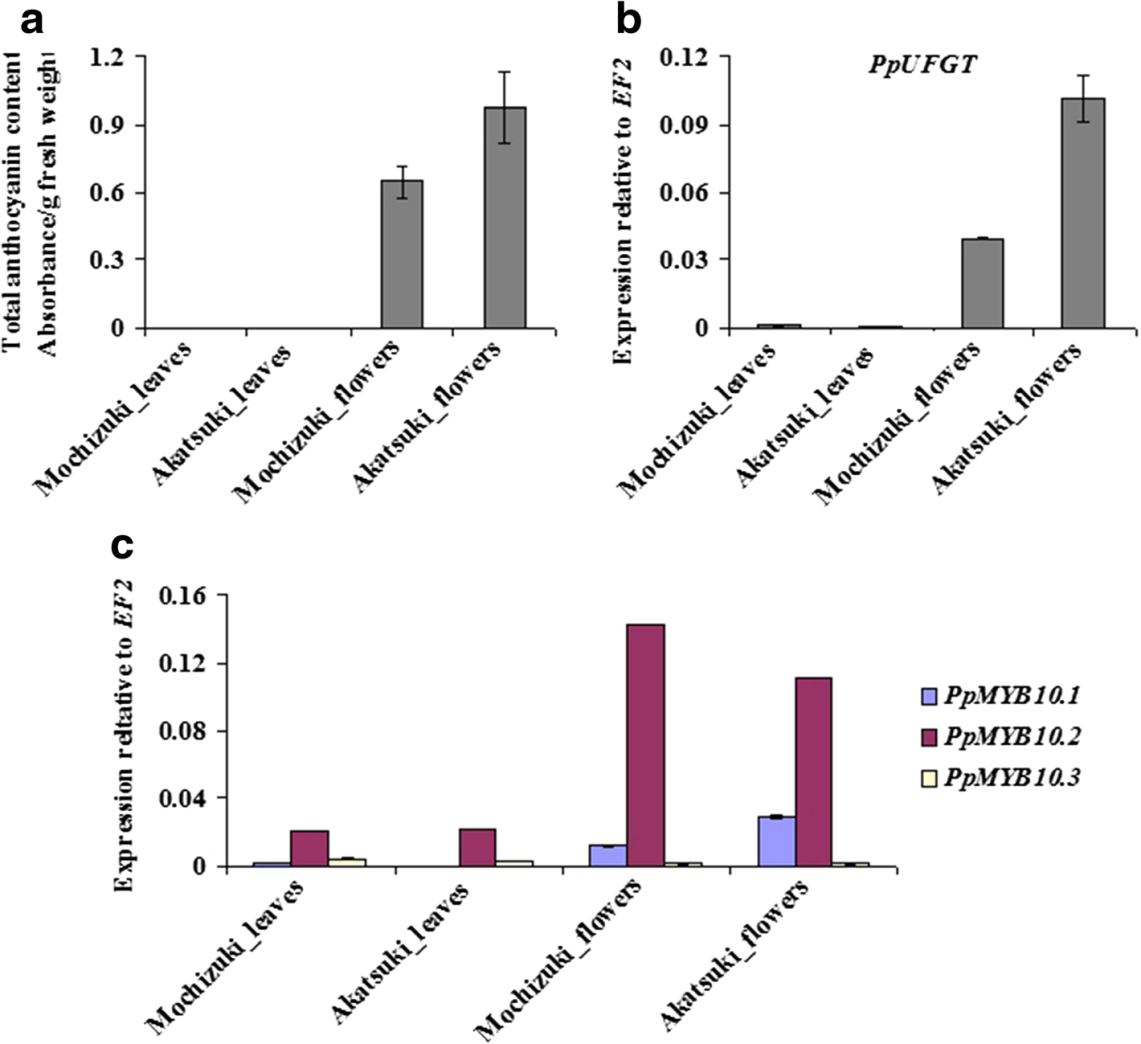
**a** Total anthocyanin content. Expression levels of *PpUFGT* (**b**) and *PpMYB10.1*/*2*/*3* (**c**) in leaves and flowers of ‘Mochizuki’ and ‘Akatsuki’. Height of *bars* and *error bars* shows the mean and standard error, respectively, from three independent measurements

Leaves of both ‘Mochizuki’ and ‘Akatsuki’ are green and showed no anthocyanin accumulation and very low *PpUFGT* transcription (Fig. 6a, b). *PpMYB10.1* and *PpMYB10.3* were also poorly transcribed, but a high expression level of *PpMYB10.2* was found in the leaves of ‘Mochizuki’ and ‘Akatsuki’ (Fig. 6c). ‘Akatsuki’ flowers showed higher *PpUFGT* expression levels than ‘Mochizuki’ flowers, leading to higher total anthocyanin content in ‘Akatsuki’ flowers (Fig. 6a, b). It was correlated with the expression of *PpMYB10.1* in flowers of ‘Mochizuki’ and ‘Akatsuki’. *PpMYB10.2* transcription was high but not associated with anthocyanin content, and only trace *PpMYB10.3* expression was detected in flowers of ‘Mochizuki’ and ‘Akatsuki’. These results indicate that *PpMYB10.1* is responsible for anthocyanin accumulation in flowers of ‘Mochizuki’ and ‘Akatsuki’. *PpMYB10.2* and *PpMYB10.*3 have functions other than anthocyanin accumulation in leaves and flowers of ‘Mochizuki’ and ‘Akatsuki’.

### Functional analysis of *PpMYB10.1* by transient promoter assay in a heterologous system

**Fig. 7:**
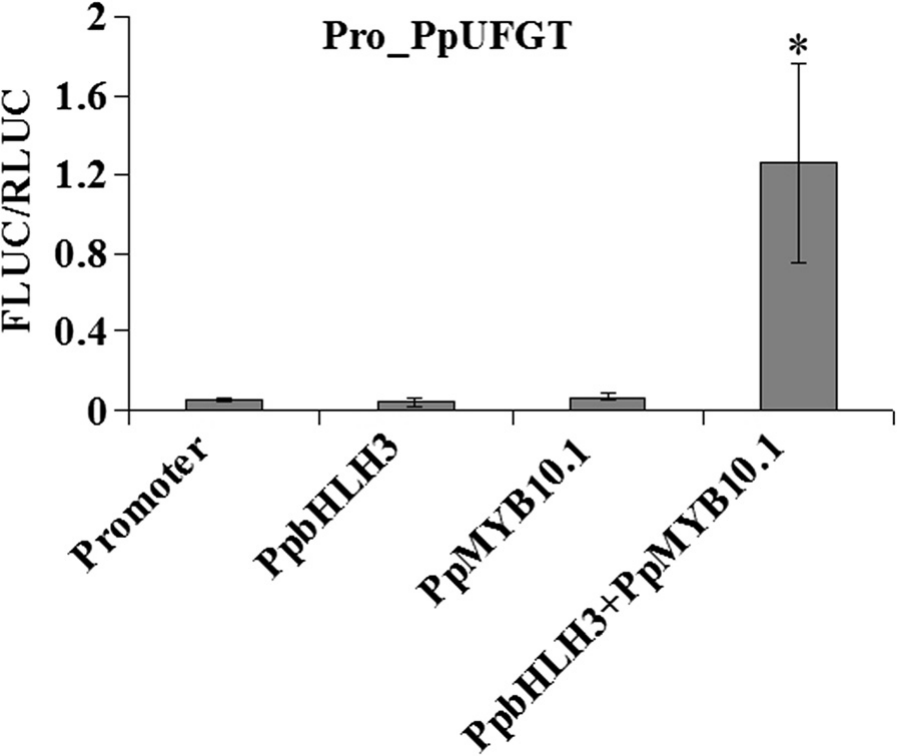
Transient activation of the 2000-bp upstream regions of *PpUFGT* by *PpMYB10.1* alone or in combination with *PpbHLH3*. Height of the *bars* and *error bars* shows the mean and standard error, respectively, from six independent measurements. The *asterisk* indicates a significant difference (*P* <0.05) from leaves infiltrated with the only promoter of *PpUFGT*, by using the two-tailed Student’s t-test

To evaluate the regulatory capacity of *PpMYB10.1* on the expression of *PpUFGT*, transient expression of a FLUC reporter gene under the control of the putative *PpUFGT* promoter regulated by *PpMYB10.1* alone or a combination of *PpMYB10.1* and *PpbHLH3* was evaluated in *Nicotiana benthamiana* leaves. As shown in Fig. 7, activity of the *PpUFGT* promoter was significantly induced by *PpMYB10.1* in the presence of *PpbHLH3. PpMYB10.1* and *PpbHLH3* alone could not significantly increase the promoter activity of *PpUFGT*.

### Investigation of the differences in *PpMYB10.1* expression in ‘Mochizuki’ and ‘Akatsuki’

**Fig. 8:**
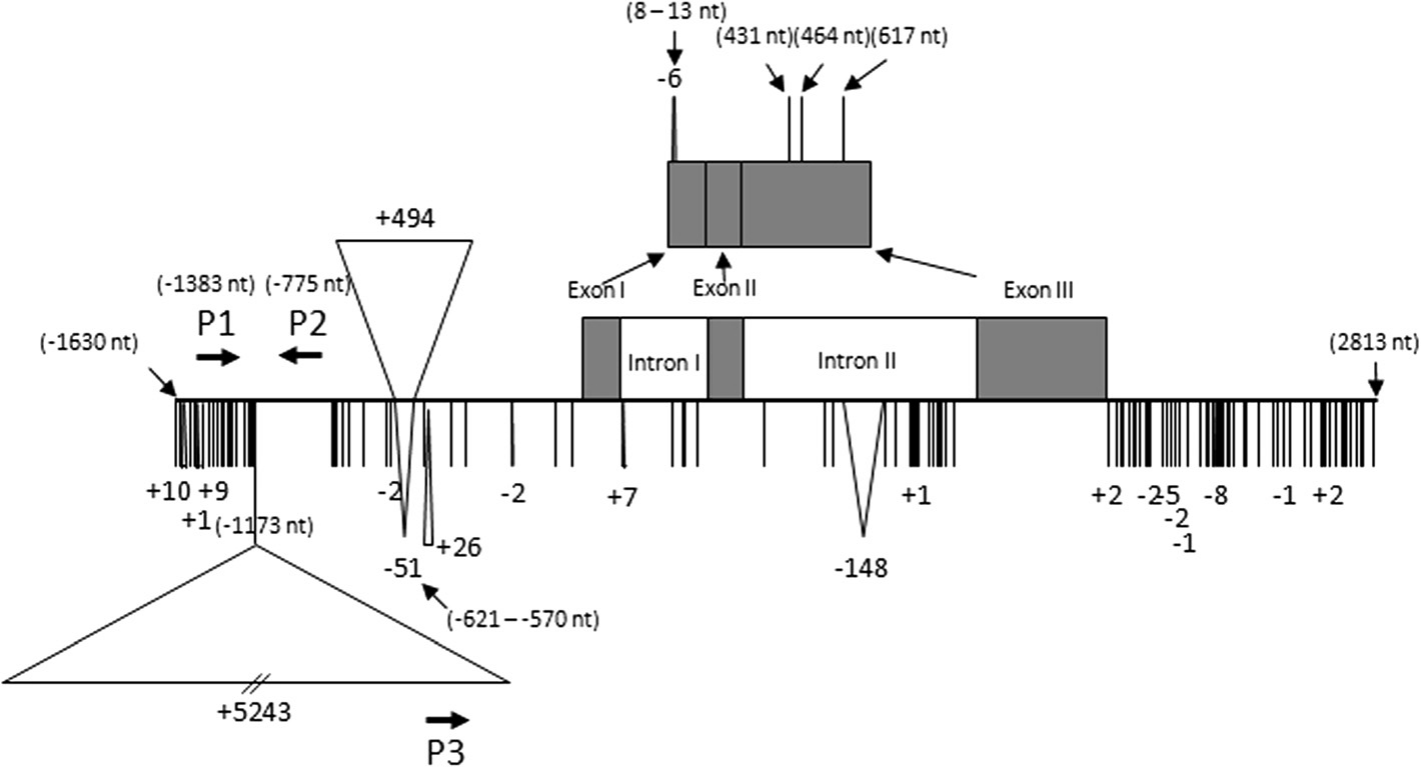
Genomic structure of *PpMYB10.1* allele. “∣”, “+n”, and “-n” indicate the single nucleotide polymorphism, number of nucleotide insertion, and number of nucleotide deletion, respectively, of MYB10.1-2 type compared to MYB10.1-1 type. (n nt) indicate the nucleotide position relative to start codon ATG. P1, P2, and P3 are three primers used to discriminate MYB10.1 allelic types

Since *PpMYB10.1* was differentially expressed in red-skinned ‘Akatsuki’ and white-skinned ‘Mochizuki’, we then intended to investigate the expression of *NAC* including *Blood* (*PpBL*) and *SQUAMOSA promoter-binding protein-like transcription factor* (*PpSPL1*) genes that have been reported as upstream transcription factors for *MYB* regulation in red-fleshed peach [27]. The expression level of *PpBL* in ‘Akatsuki’ was higher than in ‘Mochizuki’, but apparent expression of *PpBL* was also recorded even in white-skinned ‘Mochizuki’ albeit less much (Additional file 3: Figure S3). Expression of *PpSPL1*, which was believed as a transcriptional repressor of the promoter of *PpMYB10.1* [27], was inversely correlated with the transcription of *PpMYB10.1* in fruit skin during fruit development of ‘Mochizuki’ and ‘Akatsuki’. These results indicated that *PpMYB10.1* expression and red skin color was regulated by *PpBL* and *PpSPL1* as in the case of blood flesh [27]. However, it was noticed that *PpBL* expression was increased in skin of stage 5 of ‘Mochizuki’ fruits where *PpMYB10.1* expression level remained low, indicating other factors such as genomic structure may be also involved in the regulation of *PpMYB10.1* activity. Therefore, we further investigated the genomic sequences of *PpMYB10.1* in red-skinned cultivar and white-skinned cultivar. There were many insertion/deletion (InDel) and single nucleotide polymorphisms (SNPs) in the sequences of up- and down-streams as well as introns in Mochizuki-type PpMYB10.2 compared to Akatsuki-type PpMYB10.1 (tentatively designated as MYB10.1-1 for Akatsuki-type and MYB10.1-2 for Mochizuki-type, respectively) (Fig. 8). Especially, the coding regions of MYB10.1-2 type showed one deletion at 8–13 nt of exon I and three SNPs at 431, 464 and 617 nt of exon III, causing two amino acid deletion and four amino acid substitution in coding regions of MYB10.1-2 compared to that of MYB10.1-1 type (Additional file 4: Figure S4). Based on these three SNPs of exon III, we investigated the mRNA sequences of *PpMYB10.1* in ‘Shimizu-hakuto’, which was preliminary found to be heterozygous for *PpMYB10.1* locus (MYB10.1-1/MYB10.1-2). The results showed that all the transcripts of *PpMYB10.1* from 60 independent clones were derived from MYB10.1-1 only (Table 1), indicating that the reason for induction of *PpMYB10.1* expression is ascribed to *PpMYB10.1* genomic structure. Based on the differences in two *PpMYB10.1* types, we tried to classify 23 Japanese peach cultivars. Thirteen cultivars showing red skin color (color index = 2, Additional file 5: Figure S5a) including ‘Akatsuki’ possess two MYB10.1-1 type, while those with white skin color (color index = 0, Additional file 5: Figure S5b) including ‘Mochizuki’ had two MYB10.1-2 type (Table 2, Additional file 6: Figure S6). Among seven analyzed cultivars with pale red skin color (color index = 1, Additional file 5: Figure S5c), three cultivars had two MYB10.1-1 type, while other four cultivars were consisted of one MYB10.1-1 type and one MYB10.2 type (Table 2, Additional file 6: Figure S6). Although we cannot exclude other possibilities such as epigenetic regulation for the differences in *PpMYB10.1* expression in ‘Mochizuki’ and ‘Akatsuki’, these results demonstrated that the skin color phenotypes can be partially explained by the differences in *PpMYB10.1* allelic types.

**Table 1:** Sequence analysis of *PpMYB10.1* transcript in *PpMYB10.1*-heterozygous ‘Shimizu-hakuto’

Type	SNPs			Number of cDNA clone
	431 nt	464 nt	617 nt	
MYB10.1-2	C	C	C	0
MYB10.1-1	T	A	G	60

**Table 2:** Classification of 23 Japanese peach cultivars according to red color index and *PpMYB10.1* allele

Color index	Cultivar	PpMYB10.1 allele
0	Mochizuki, Hanashimizu, and Yamate-shimizu	MYB10.1-2/MYB10.1-2
1	Shimizu-hakuto, Yamato-hakuto, Shimizu-hakuto RS, and Hakushu	MYB10.1-1/MYB10.1-2
	Hakuto, Setouchi-hakuto, and Sakigake-hakuto	MYB10.1-1/MYB10.1-1
2	Akatsuki, Hakuho, Hanayome, Hikawa-hakuho, Kanouiwa-hakuto, Kawanakajima-hakuto, Hakurei, Natsugokoro, Misakakko, Benishimizu, Asama-hakuto, Chikusa-hakuto, and Tenshin suimitsu	MYB10.1-1/MYB10.1-1

## Discussion

### *PpMYB10.1* contributes to anthocyanin accumulation in peach fruit skin and flowers

The transcription factor MYB10 group has been reported to be responsible for the red coloration in the skin of several Rosaceae fruit trees such as apple, pear, and strawberry [21, 23, 28]. For peach, Ravaglia et al. [25] first demonstrated the importance of *PpMYB10* (corresponding to *PpMYB10.2*) in anthocyanin accumulation in nectarine, while Rahim et al. [26] reported that expression levels of *PpMYB10.1* and *PpMYB10.3* correlated with the anthocyanin content of the peel, mesocarp, and mesocarp around the stone. These results were not consistent with our expression analysis results that *PpMYB10.2*/*3* transcript was very low in the red-skinned samples and only expression of *PpMYB10.1* was highly consistent with anthocyanin accumulation throughout fruit development (Figs. 2b and 3b). Recently, Zhou et al. [27] also showed that only *PpMYB10.1* expression is correlated with anthocyanin level in blood-flesh peach. We assumed that the anthocyanin biosynthetic mechanism may depend on the analyzed peach cultivars and fruit tissues. In this study, the direct contribution of *PpMYB10.1* to anthocyanin accumulation and red coloration of fruit skin and flowers of ‘Akatsuki’ was found (Figs. 2b, 3b and 6). During fruit development in ‘Mochizuki’ and ‘Akatsuki’, *PpMYB10.1* expression was highly correlated with *PpUFGT* (Fig. 2c). Transient expression analysis of tobacco leaves indicated that *PpMYB10.1*/*PpbHLH3* significantly increased the activities of the *PpUFGT* promoter (Fig. 7). Furthermore, overexpression of *PpMYB10.1* resulted in higher anthocyanin content and deeper red color in transgenic tobacco and increased only *NtUFGT* expression but no other branching genes in the flavonoid biosynthetic pathway (Fig. 5b, Additional file 2: Figure S2). In addition, *PpMYB10.1* alone contributes to anthocyanin accumulation and red coloration in the flesh, like in the skin of ‘Akatsuki’ fruit (Additional file 7: Figure S7). These results confirm that *PpMYB10.1* is the key regulator of anthocyanin biosynthesis and that it successfully activates the promoter of *PpUFGT* at least in Japanese peach cultivars, including ‘Akatsuki’.

### *PpMYB10.2*/*3* contributes to processes other than anthocyanin accumulation

Expression levels of *PpMYB10.2*/*3* were low throughout the fruit development of ‘Mochizuki’ and ‘Akatsuki’ and were not associated with anthocyanin accumulation and red coloration (Fig. 3b). A considerable level of *PpMYB10.2* expression was detected in peach leaves that do not contain anthocyanin, and high expression of *PpMYB10.2* was not also consistent with the anthocyanin content of flowers of ‘Mochizuki’ and ‘Akatsuki’ (Fig. 6). Rahim et al. [26] showed that *PpMYB10.2* contributes to anthocyanin accumulation during leaf senescence and flower development. However, a recent study indicated that *PpMYB10.4* on linkage group 6 regulates anthocyanin accumulation in peach leaf, while *PpMYB10.2* do not contribute to the leaf red coloration [29]. In this study, our results also proposed that *PpMYB10.2*/*3* do not play roles in the accumulation of anthocyanin in peach leaves and flowers (Fig. 6). Moreover, introduction of *PpMYB10.2*/*3* did not alter the flavonoid biosynthetic genes in tobacco as well anthocyanin accumulation in transgenic tobacco flowers (Fig. 4, Additional file 2: Figure S2). In *Arabidopsis*, *AtMYB75* (NM_104541) and *AtMYB90* (AF062915), named *Production of Anthocyanin Pigment 1* (*AtPAP1*) and *2* (*AtPAP2*), respectively, were identified as regulators of anthocyanin biosynthesis. *AtPAP1* and *AtPAP2* share high sequence identity and were clustered together in the MYB phylogeny tree constructed by Ravaglia et al. [25]. However, overexpression of not *AtPAP2* but *AtPAP1* stimulates expression level of the anthocyanin structural gene and anthocyanin accumulation in seedlings of transgenic *Arabidopsis* plant [30]. Similarly, *PpMYB10.1*/*2*/*3* share high sequence identity at amino acid level. We hypothesized that the R/B-like bHLH binding motifs ([DE]Lx_2_[RK]x_3_Lx_6_Lx_3_R) of *PpMYB10.2*/*3* showed an amino acid different with those of *PpMYB10.1* (Fig. 3a) [31], by which *PpMYB10.2*/*3* proteins probably act with different bHLHs with *PpMYB10.1* to play roles in processes other than anthocyanin biosynthesis.

### Preliminary comparisons of *MYB10.1* alleles of white-skinned and red-skinned peach cultivars

Since *PpMYB10.1* is a key regulatory gene for anthocyanin accumulation in skin, flower, and flesh, we tried to obtain a preliminary insight into the differences of *PpMYB10.1* activation observed in ‘Mochizuki’ and ‘Akatsuki’. We first tried to find the cause in the upstream transcription factors of *PpMYB10.1.* Expression analysis of *PpSPL1* and *PpNAC* indicated that these genes could be one of the factors for regulating *PpMYB10.1* expression levels in ‘Mochizuki’ and ‘Akatuski’, but other factors would be also involved in the cause for differential expressions observed (Fig. 3b). To confirm this, we identified the transcribed *PpMYB10.1* types using ‘Shimizu-hakuto’. The results clearly showed that all the transcribed *PpMYB10.1* types were derived from not MYB10.1-2 but MYB10.1-1 (Table 1), indicating that the activation of *PpMYB10.1* expression depends on *PpMYB10.1* allelic types. We then compared the differences in nucleotide sequences of *PpMYB10.1* ORFs between white-skinned ‘Mochizuki’ and red-skinned ‘Akatsuki’ peach, causing different *PpMYB10.1* proteins were translated from MYB10.1-1 and MYB10.1-2 types (Additional file 4: Figure S4). Moreover, several sequence differences in MYB10.1-1 and MYB10.1-2 including InDels and SNPs were recorded in the 5′-upstream, 3′-downstream, and intron (Fig. 8). Thus, genomic sequences of *PpMYB10.1* using 23 Japanese peach cultivars with being different skin color enabled to classify into two types of PpMYB10.1, MYB10.1-1 and MYB10.1-2 type, where two MYB10.1-2 types resulted in the white skin color, while two MYB10.1-1 or MYB10.1-1/MYB10.1-2 types showed red or pale red skin color (Table 2). The question that which sequence differences among MYB10.1-1 and MYB10.1-2 types are the cause for activation and inactivation of *PpMYB10.1* was raised. Zhou et al. [27] who surveyed the differences in DNA sequences of *PpMYB10.1* between Chinese white-fleshed and red-fleshed peach cultivars, reported a 483-bp insertion and a SNP in the promoter region and the second intron of ‘Dahongpao’ blood-fleshed cultivar, respectively. This 483-bp insertion seems to be comparable to the 494-bp InDel found in our study (Fig. 8). However, this 483-bp insertion was not proved to link to the red flesh color in other Chinese peach cultivars [27]; in other word, this insertion was not a cause for activation/inactivation of *PpMYB10.1*. Similarly, considering that peach reference MYB10.1 gene using red peach cultivar ‘Lovell’ possessed this insertion (see https://www.rosaceae.org/species/prunus/prunus_persica), we also assumed that 494-bp insertion in MYB10.1-2 did not relate to the function, but our 494-bp InDel was found to associate with the red color index in 23 Japanese cultivars. Twenty-three Japanese peach cultivars examined in this study share close genetic relationships to each other as you can see the several sports of ‘Hakuto’ peach (Table 2), which may explain why *PpMYB10.1* allelic types were associated with the red color indexes in skin of these cultivars.. Therefore, we are not sure if our results can apply to other peach cultivars/strains especially other than Japanese cultivars. By contrast, we also found other InDels and many SNPs in the 5′-upstream, 3′-downstream, exons and introns in *PpMYB10.1* alleles of Japanese peach cultivars. It suggested that the genomic structures of Japanese peach cultivars are not completely similar with those of Chinese peach cultivars [27]. At present, we have not yet determined the actual key sequence differences in MYB10.1-1 and MYB10.1-2 types that lead to the activation of MYB10.1-1 type. Further surveys are required to establish the essential factors in DNA sequences that directly contributed to the expression of *PpMYB10.1* in red-skinned peach.

## Conclusions

We concluded that *PpMYB10.1* is a major regulator of anthocyanin accumulation in red-skinned peach and that a close relationships between *PpMYB10.1* allelic type and skin color phenotype (at least in 23 Japanese cultivars). This finding will enable further investigation of DNA sequences of *PpMYB10.1*, providing essential information to establish DNA markers linked to white- and red-skinned peach for fruit breeders.

## Methods

### Plant materials

Peach fruit were collected from ‘Mochizuki’ and ‘Akatsuki’ trees grown in the orchard of the NARO Institute of Fruit Tree Science, Tsukuba, Japan (latitude 36°N, longitude 140°E). Leaves and flowers were collected on April 26, 2013. Peach skin and flesh were separated manually from three to nine fruit at five different stages of fruit development (Additional file 8: Figure S8). Other cultivars shown in Table 2 were cultivated in the orchard of the Okayama Prefectural Technology Center for Agriculture, Forestry, and Fisheries, Okayama, Japan (latitude 35 °N, longitude 134 °E). The skin samples were frozen in liquid nitrogen and stored at –80 °C until analysis. We classified 23 peach cultivars based on the degree/intensity of red pigmentation in the peach skin; we defined color index 0, 1 and 2 as white skin color, pale red skin color and red skin color, respectively (see Additional file 5: Figure S5).

### qRT-PCR and expression analysis

The peach samples were ground in a mortar with liquid nitrogen, and total RNA was extracted from the frozen powder by using the hot-borate method [32]. Gel electrophoresis and spectrophotometry were performed to test the quality and concentration of the extracted RNA, respectively. For first-strand cDNA synthesis, 1 μg of high-quality total RNA was used for reverse transcription with the SuperScript VILO cDNA Synthesis Kit (Invitrogen, Carlsbad, CA, USA). A 20-fold dilution of the 20-μl cDNA was used as the template for qRT-PCR. Target genes were shown in Additional file 9: Table S1. RT-PCR primers were designed using the Primer3 website (http://bioinfo.ut.ee/primer3-0.4.0/primer3/, Additional file 9: Table S1). RT-PCR products were confirmed by fragment sizes, melting curves, and sequencing. Expression levels of target genes were analyzed by relative quantification, with the *elongation factor 2* housekeeping gene (EF2, ppa001368m) as the reference gene. The reaction mixture (10 μl) contained 1 μl of diluted cDNA sample, 0.5 μl of each primer (10 μM), 5 μl of GeneAce SYBR qPCR Mix α Low ROX (Nippon Gene, Tokyo, Japan), and DEPC water. qRT-PCR was performed using the 7500 Real Time PCR System (Applied Biosystems, Foster City, CA, USA), and the results were analyzed with the 7500 System Sequence Detection Software ver. 1.4. Three biological replications with three technical replications for each sample were used for real-time analyses.

### Plasmid vector construction for transformation

ORFs of *PpMYB10.1*/*2*/*3* were generated from first-strand cDNA of ‘Akatsuki’ by using PCR with KOD-Neo DNA polymerase (Toyobo, Osaka, OSK, Japan). Purified PCR fragments with the expected size bands were cloned into the pENTR/D-TOPO vector (Invitrogen), according to the manufacturer’s instructions. Subsequently, the ORFs of *PpMYB10.1*/*2*/*3* were inserted into the pGWB2 vector under transcriptional control of the 35S CaMV promoter [33] by using LR clonase (Invitrogen). Recombinant pGWB2 plasmids were then transferred into *Agrobacterium tumefaciens* LBA4404 by using the heat-shock method and grown at 28 °C on Luria-Bertani medium with kanamycin, hygromycin, and rifampicin.

### Stable transformation of *N. tabacum*

Excised leaves of *N. tabacum* SR1 from 4-week-old seedlings were used as the explant material for co-cultivation with *A. tumefaciens* LBA4404 harboring *PpMYB10.1/2/3* overexpression constructs. The transformation was carried out as described previously by Dobhal et al. [34]. Flowers of 3-month-old transgenic tobacco plants were collected and immediately frozen in liquid nitrogen and then stored at −80 °C for RNA isolation and anthocyanin analysis. Total RNA was extracted from the transgenic tobacco flowers by using cetyltrimethylammonium bromide buffer and a silica column-based extraction method [35]. Expression levels of *NtFLS* (AB289451), *NtANR* (AM791704), *NtLAR* (AM827419), *NtUFGT* (GQ395697), and transgenes *PpMYB10.1*/*2*/*3* were analyzed using *NtActin* (X69885) as the reference. Primers used for gene expression analysis of transgenic tobacco are listed in Additional file 9: Table S1.

### Transient dual-luciferase assay

The putative promoter region of peach *PpUFGT*, which is 2000 bp upstream from the start codon, was amplified using genomic DNA extracted from “Akatsuki” fruit (primers are listed in Additional file 9: Table S1). The promoter region of *PpUFGT* was cloned into pGWB35 [33], in which the firefly luciferase (FLUC) gene is under the control of the cloned promoters. As the internal standard vector, renilla luciferase (RLUC) gene amplified from the pRL-SV40 vector (Promega, Madison, WI, USA) was cloned into the pBGW2 vector. ORFs of *PpMYB10.1* and *PpbHLH3* (ppa002884m) amplified from “Akatsuki” first-strand cDNA were also cloned into pGWB2. All resultant plasmids were transformed into *A. tumefaciens* LBA4404. Transient expression assays were performed using *N. benthamiana* leaves with the method available in our laboratory [36]. FLUC and RLUC were assayed using dual luciferase assay reagents (Promega, Madison, WI, USA), and their activities were detected with the ChemiDoc™ MP System (Bio Rad, Hercules, CA, USA). Data were collected as the ratio of FLUC to RLUC. Six independent biological replications (six leaves from different plants) and technical replications (2–3 injections of the same leaf) were performed for each treatment.

### Measurements of total anthocyanin

The peach and transgenic tobacco samples were placed in 10 ml of methanol:hydrochloric acid (99:1, v/v). Extractions were performed overnight in the dark at 4 °C. Absorbance of each extract was measured at 530, 620 and 650 nm by using a spectrophotometer (UV-2450; Shimadzu, Kyoto, Japan). Relative anthocyanin content was normalized using the following formula: Normalized OD530 = [(OD530-OD650) – 0.2 × (OD650-OD620)] [37]. Three independent biological replicates were performed for each measurement.

### Analysis of *PpMYB10.1* alleles transcribed in ‘Shimizu-hakuto’ skin

The skin of ‘Shimizu-hakuto’ whose *PpMYB10.1* allelic types were preliminary analyzed as MYB10.1-1 and MYB10.1-2 types, were frozen by liquid nitrogen and subsequently powdered using a Multi Beads Shocker (Yasui Kikai, Osaka, Japan). Total RNA was extracted from powdered skin with cetyltrimethylammonium bromide method [38]. For first-strand cDNA synthesis, 1 μg of high-quality total RNA was used for reverse transcription with the SuperScript VILO cDNA Synthesis Kit (Invitrogen, Carlsbad, CA, USA). RT-PCR primers for discriminating *PpMYB10.1* allelic types were shown in Additional file 9: Table S1. RT-PCR products were subcloned into Zero Blunt TOPO PCR cloning kit (Invitrogen), and sequencing.

### Analysis of genomic sequences of *PpMYB10.1* in 23 peach cultivars

We then determined the genomic sequences using 23 Japanese peach cultivars (Table 2). Genomic DNA from peach skin was extracted using the DNeasy Plant Mini Kit (Qiagen, Tokyo, Japan). We first carried out PCR using genomic DNA with the primers for discriminating the InDel using P1, P2, and P3 primer sets (primers in Additional file 9: Table S1). Briefly, PCR was carried out with the mixtures of 3 primers, where the band of either 609 or 426 bp was amplified in case of MYB10.1-1/MYB10.1-1 (by P1/P2) or MYB10.1-2/MYB10.1-2 (P2/P3), respectively, while both bands were amplified for MYB10.1-1/MYB10.1-2. Here, the combination of P1/P2 primer could not generate band in MYB10.1-2/MYB10.1-2 types due to the long size (over 5800 bp) of amplicon. Then, the sequences in the 5′-upstream, 3′-downstream, exon and intron were obtained by PCR using several primer sets (data not shown). PCR products were subcloned and sequenced as aforementioned.

## Availability of supporting data

All supporting data are included as additional files.

## Additional files

Additional file 1: Figure S1.
**(a)** Confirmation of the presence of transgenes *PpMYB10.1*/*2*/*3* in the transgenic tobacco genome by using the CaMV 35S primer (5′-TCCACTGACGTAAGGGATGAC-3′) and PpMYB10.1/2/3 ORF reverse primers. POS, positive control; WT, wild-type tobacco plant; #n, independent line numbers. (**b**) Red coloration in capsule skin of transgenic tobacco plants overexpressing *PpMYB10.1*. (JPG 2499 kb) Additional file 2: Figure S2. Expression levels of branching genes involved in the flavonoid biosynthetic pathway – *NtFLS*, *NtLAR*, and *NtANR* in transgenic tobacco flowers. Height of the bars and error bars shows the mean and standard error, respectively, from three independent measurements. (JPG 646 kb) Additional file 3: Figure S3. Expression levels of *PpBL* and *PpSPL1* in the skin of ‘Mochizuki’ and ‘Akatsuki’ during fruit development. Height of bars and error bars shows the mean and standard error, respectively, from three independent measurements. (JPG 49 kb) Additional file 4: Figure S4. Amino acid alignments of MYB10.1-1 and MYB10.1-2 ORFs amplified from first-strand cDNA of ‘Shimizu-hakuto’. (JPG 83 kb) Additional file 5: Figure S5. Photographs of peach fruits classified to red color index 0 (**a**) ‘Hanashimizu’, 1 (**b**) ‘Shimizu-hakuto’, and 2 (**c**) ‘Hakuho’. (JPG 1479 kb) Additional file 6: Figure S6. Analysis of MYB10.1 alleles in 23 Japanese peach cultivars using P1, P2, and P3 primers. ‘Akatsuki’, ‘Hakuho’, ‘Hanayome’, ‘Hikawa-hakuho’, ‘Kanouiwa-hakuto’, ‘Kawanakajima-hakuto’, ‘Hakurei’, ‘Natsugokoro’, ‘Misakakko’, ‘Benishimizu’, ‘Asama-hakuto’, ‘Chikusa-hakuto’, ‘Tenshin suimitsu’, ‘Hakuto’, ‘Setouchi-hakuto’, and ‘Sakigake-hakuto’ showed 609-bp bands (MYB10.1-1/MYB10.1-1). ‘Mochizuki’, ‘Hanashimizu’, and ‘Yamate-shimizu’ showed 426-bp bands (MYB10.1-2/MYB10.1-2). ‘Shimizu-hakuto’, ‘Yamato-hakuto’, ‘Shimizu-hakuto RS’, and ‘Hakushu’ had both bands (MYB10.1-1/MYB10.1-2). (JPG 108 kb) Additional file 7: Figure S7. ‘Akatsuki’ usually shows white flesh, but can accumulate anthocyanin to some extent depending on the ripening stages and environmental conditions, which are not yet fully addressed. In contrast, ‘Mochizuki’ seldom shows red pigmentation in its flesh. (**a**) Photographs of fruit flesh. (**b**) Total anthocyanin content. (**c**) Expression levels of *PpMYB10.1*/*2*/*3* in the flesh of ‘Mochizuki’ and ‘Akatsuki’ during fruit development. Height of bars and error bars shows the mean and standard error, respectively, from three independent measurements. (JPG 751 kb) Additional file 8: Figure S8. Weights (**a**) and sizes of equatorial diameters (**b**) of ‘Mochizuki’ and ‘Akatsuki’ fruit harvested on May 17 (S1), June 03 (S2), June 27 (S3), July 10 (S4), and July 24 (S5), 2013. (JPG 901 kb) Additional file 9: Table S1. Primers used in this study. (DOCX 28 kb)

## Abbreviations

ANS: anthocyanidin synthase
bHLH: basic helix-loop-helix
BL: blood
CHI: chalcone isomerase
CHS: chalcone synthase
DFR: dihydroflavonol-4-reductase
F3H: flavanone 3-hydroxylase
FLUC: firefly luciferase
ORF: open reading frame
qRT_PCR: quantitative real-time polymerase chain reaction
RLUC: renilla luciferase
SPL1: SQUAMOSA promoter-binding protein-like transcription factor
UFGT: UDP-glucose:flavonoid-3-*O*-glucosyltransferase
