# Supplementary figures and images for: The crucial role of PpMYB10.1 in anthocyanin accumulation in peach and relationships between its allelic type and skin color phenotype
Source: BMC Plant Biol. 2015 Nov 18;15:280. doi: 10.1186/s12870-015-0664-5 (PMC4652394; doi:10.1186/s12870-015-0664-5)

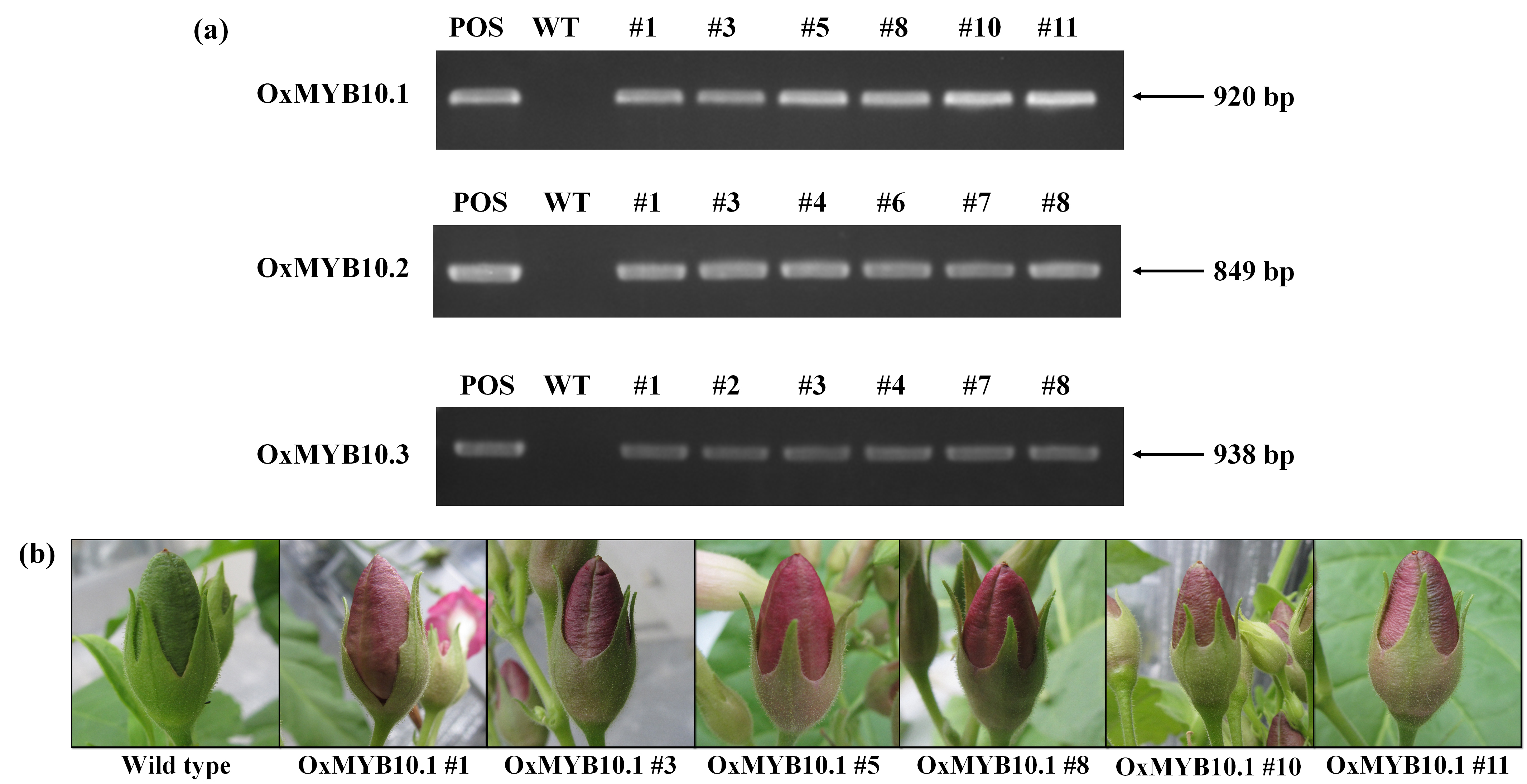

Supplement: Additional file 1: Figure S1. — (a) Confirmation of the presence of transgenes PpMYB10.1/2/3 in the transgenic tobacco genome by using the CaMV 35S primer (5′-TCCACTGACGTAAGGGATGAC-3′) and PpMYB10.1/2/3 ORF reverse primers. POS, positive control; WT, wild-type tobacco plant; #n, independent line numbers. (b) Red coloration in capsule skin of transgenic tobacco plants overexpressing PpMYB10.1. (JPG 2499 kb) [file 12870_2015_664_MOESM1_ESM.jpg]

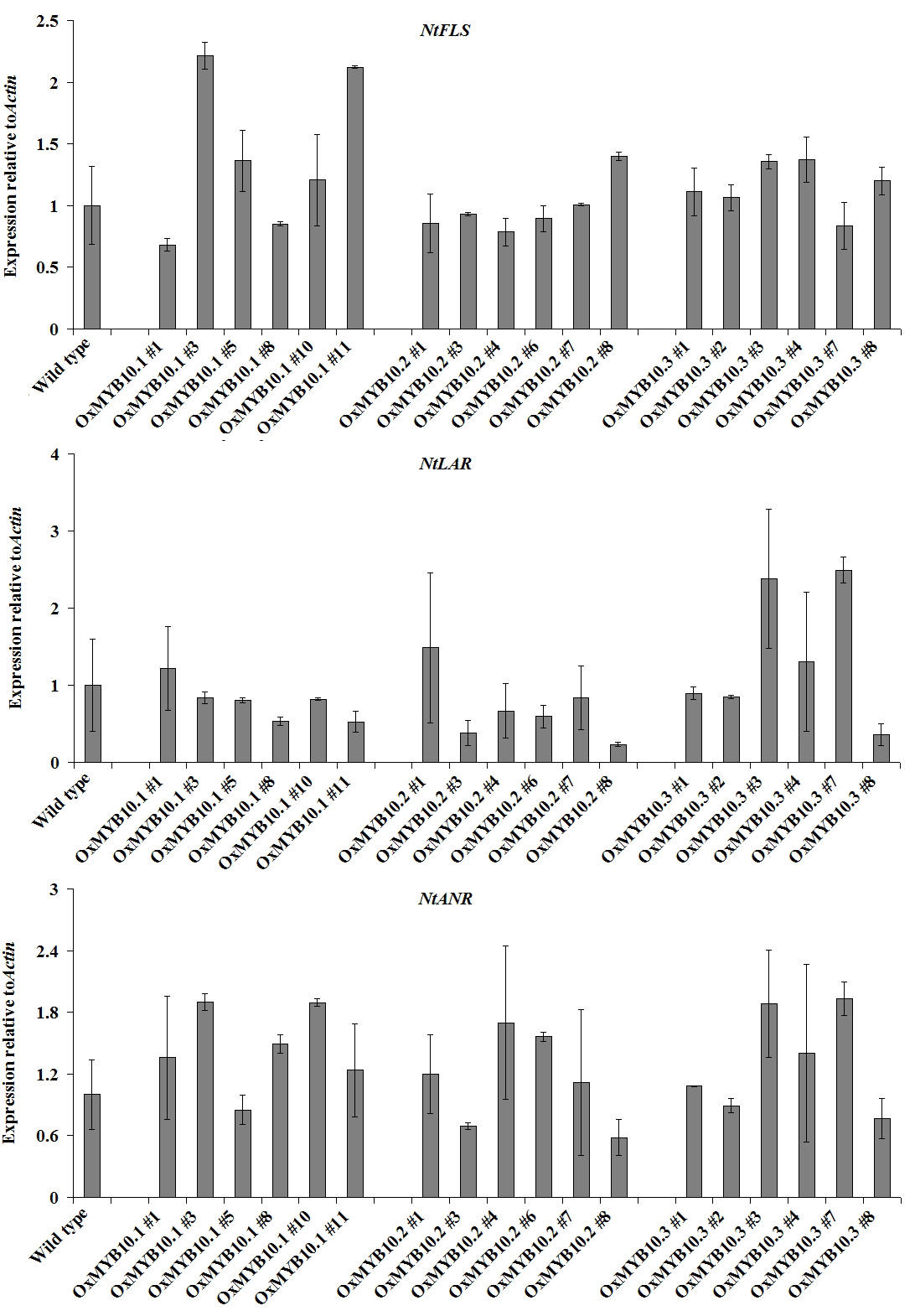

Supplement: Additional file 2: Figure S2. — Expression levels of branching genes involved in the flavonoid biosynthetic pathway – NtFLS, NtLAR, and NtANR in transgenic tobacco flowers. Height of the bars and error bars shows the mean and standard error, respectively, from three independent measurements. (JPG 646 kb) [file 12870_2015_664_MOESM2_ESM.jpg]

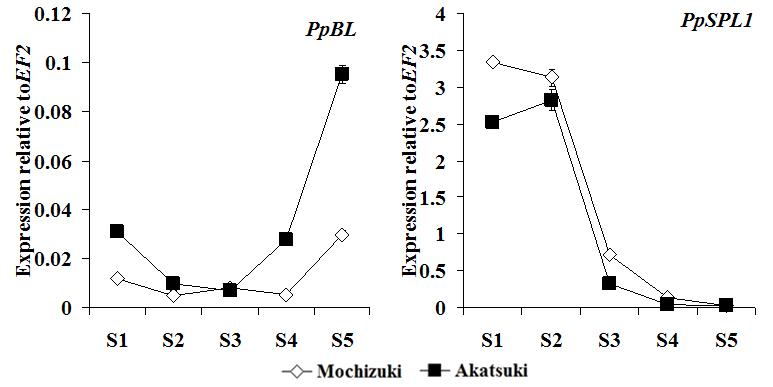

Supplement: Additional file 3: Figure S3. — Expression levels of PpBL and PpSPL1 in the skin of ‘Mochizuki’ and ‘Akatsuki’ during fruit development. Height of bars and error bars shows the mean and standard error, respectively, from three independent measurements. (JPG 49 kb) [file 12870_2015_664_MOESM3_ESM.jpg]

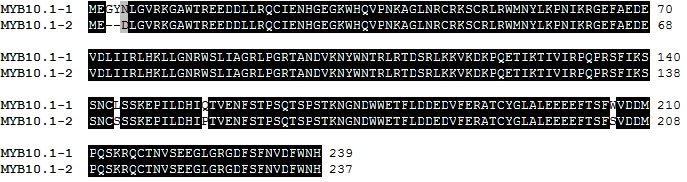

Supplement: Additional file 4: Figure S4. — Amino acid alignments of MYB10.1-1 and MYB10.1-2 ORFs amplified from first-strand cDNA of ‘Shimizu-hakuto’. (JPG 83 kb) [file 12870_2015_664_MOESM4_ESM.jpg]

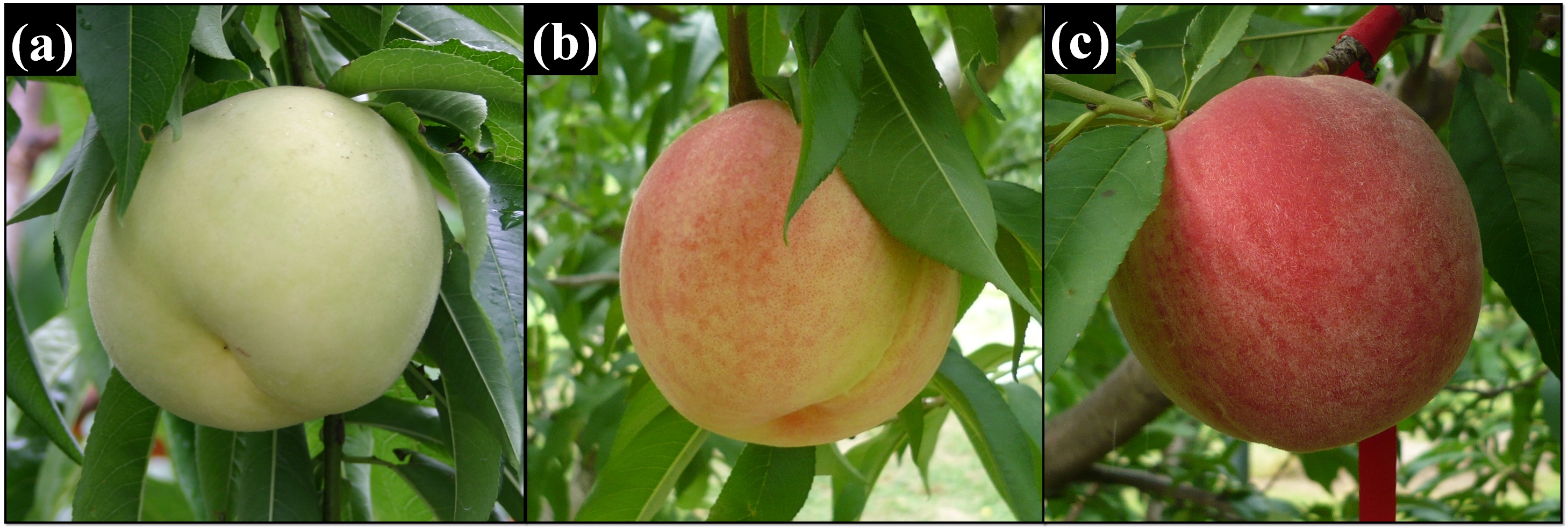

Supplement: Additional file 5: Figure S5. — Photographs of peach fruits classified to red color index 0 (a) ‘Hanashimizu’, 1 (b) ‘Shimizu-hakuto’, and 2 (c) ‘Hakuho’. (JPG 1479 kb) [file 12870_2015_664_MOESM5_ESM.jpg]

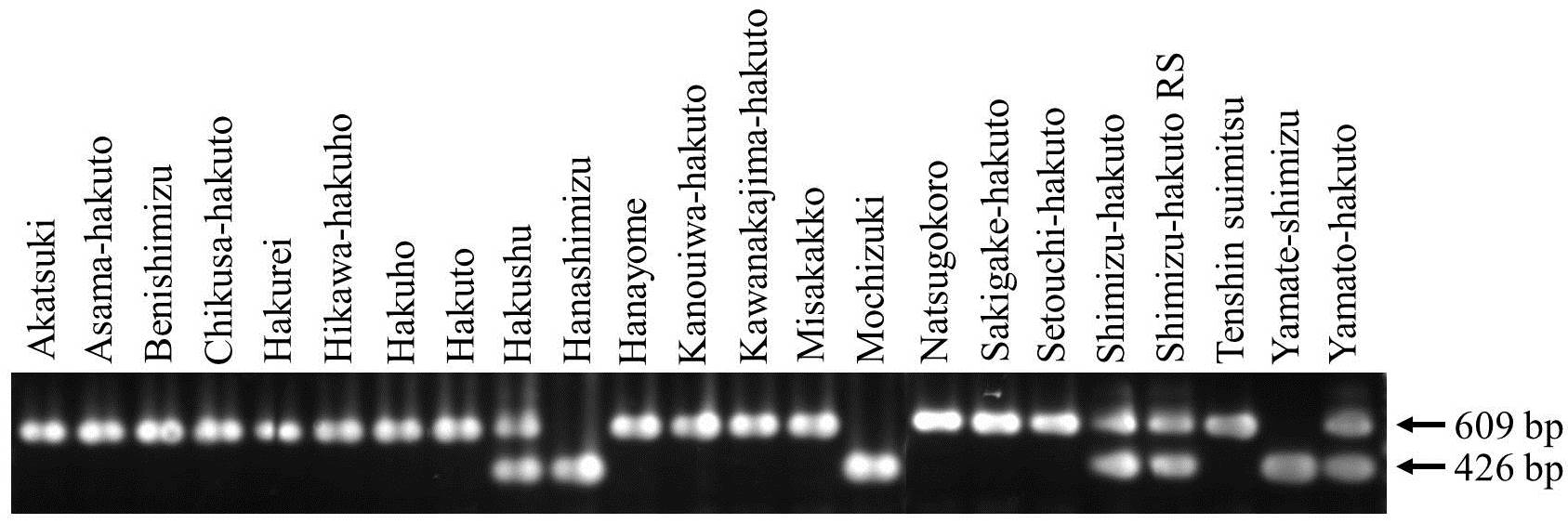

Supplement: Additional file 6: Figure S6. — Analysis of MYB10.1 alleles in 23 Japanese peach cultivars using P1, P2, and P3 primers. ‘Akatsuki’, ‘Hakuho’, ‘Hanayome’, ‘Hikawa-hakuho’, ‘Kanouiwa-hakuto’, ‘Kawanakajima-hakuto’, ‘Hakurei’, ‘Natsugokoro’, ‘Misakakko’, ‘Benishimizu’, ‘Asama-hakuto’, ‘Chikusa-hakuto’, ‘Tenshin suimitsu’, ‘Hakuto’, ‘Setouchi-hakuto’, and ‘Sakigake-hakuto’ showed 609-bp bands (MYB10.1-1/MYB10.1-1). ‘Mochizuki’, ‘Hanashimizu’, and ‘Yamate-shimizu’ showed 426-bp bands (MYB10.1-2/MYB10.1-2). ‘Shimizu-hakuto’, ‘Yamato-hakuto’, ‘Shimizu-hakuto RS’, and ‘Hakushu’ had both bands (MYB10.1-1/MYB10.1-2). (JPG 108 kb) [file 12870_2015_664_MOESM6_ESM.jpg]

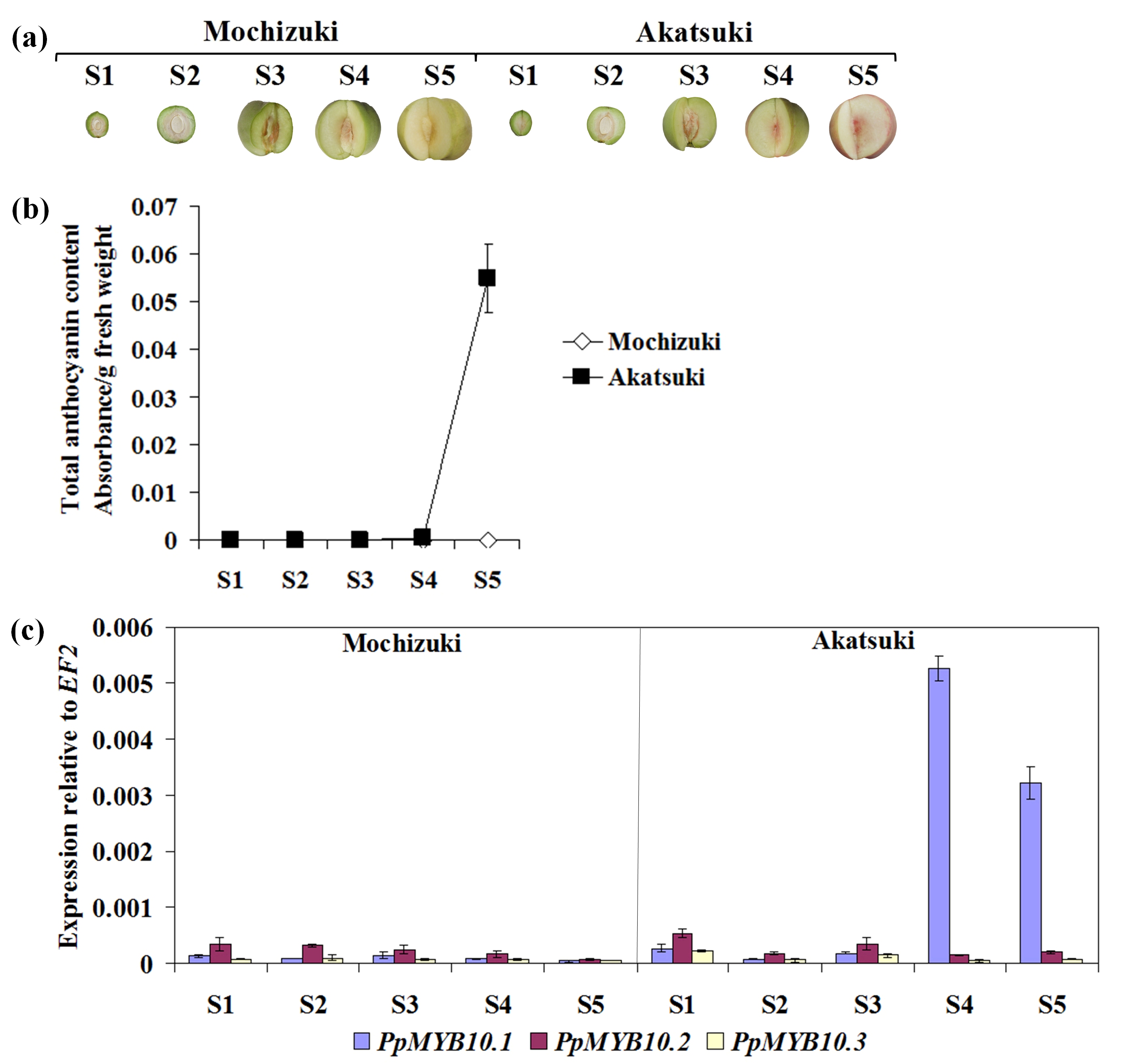

Supplement: Additional file 7: Figure S7. — ‘Akatsuki’ usually shows white flesh, but can accumulate anthocyanin to some extent depending on the ripening stages and environmental conditions, which are not yet fully addressed. In contrast, ‘Mochizuki’ seldom shows red pigmentation in its flesh. (a) Photographs of fruit flesh. (b) Total anthocyanin content. (c) Expression levels of PpMYB10.1/2/3 in the flesh of ‘Mochizuki’ and ‘Akatsuki’ during fruit development. Height of bars and error bars shows the mean and standard error, respectively, from three independent measurements. (JPG 751 kb) [file 12870_2015_664_MOESM7_ESM.jpg]

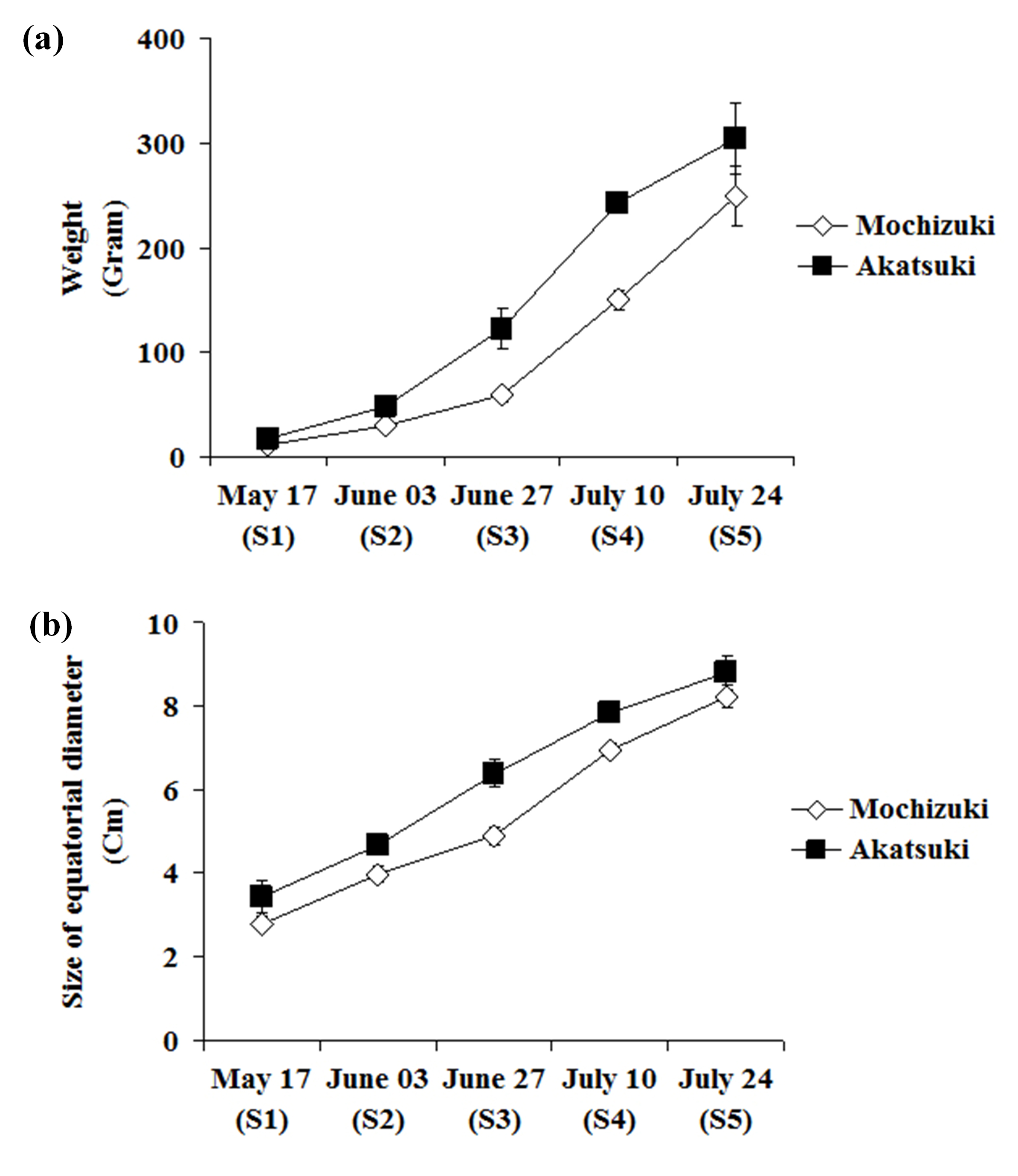

Supplement: Additional file 8: Figure S8. — Weights (a) and sizes of equatorial diameters (b) of ‘Mochizuki’ and ‘Akatsuki’ fruit harvested on May 17 (S1), June 03 (S2), June 27 (S3), July 10 (S4), and July 24 (S5), 2013. (JPG 901 kb) [file 12870_2015_664_MOESM8_ESM.jpg]
