# Supplementary material for: The crucial role of PpMYB10.1 in anthocyanin accumulation in peach and relationships between its allelic type and skin color phenotype
Source: BMC Plant Biol. 2015 Nov 18;15:280. doi: 10.1186/s12870-015-0664-5 (PMC4652394; doi:10.1186/s12870-015-0664-5)
Supplement: Additional file 9: Table S1. — Primers used in this study. (DOCX 28 kb) [file 12870_2015_664_MOESM9_ESM.docx]

**Additional file 9: Table S1.** Primers used in this study.

| Target gene  (Accession number) | Product size  (bp) | Sequences  (top: forward primer, bottom: reverse primer) |
| --- | --- | --- |
| *[Quantitative real-time PCR]* | | |
| PpMYB10.1  (ppa026640m) | 160 | GGATTCTCGCCTGAAAAAGGTG |
|  |  | CGGCGTACTAAAATTCTCGACTG |
| PpMYB10.2  (ppa016711m) | 118 | CCCAAGAAACAATAAAGACCATAA |
|  |  | GTCTCCCACCAATCACGTT |
| PpMYB10.3  (ppa020385m) | 162 | CTCTCCTGAAAACGACTACGA |
|  |  | GTGATGGCGTACTAAAATTTTCTTCTA |
| PpCHS  (ppa006888m) | 149 | AACCATCCTTCCCGACAGCGAT |
|  |  | CAGAGATACCCAAAGGTTGGAAGGC |
| PpCHI  (ppa011276m) | 142 | ACACAGGTGACAACGATACTGCCACT |
|  |  | TGAAGACCTCAAGGAACTTCTCAATGG |
| PpF3H  (ppa007636m) | 115 | TTGTGGAGGCTTGTGAGGATTGG |
|  |  | TCCGAGGGCAGAGCGAAGAAC |
| PpDFR  (ppa008069m) | 113 | ATTTCTCATGCCATCCATGCCAC |
|  |  | GGTCGTCCAAGTGAACGAACTGCC |
| PpANS  (ppa007738m) | 150 | GGAGTTGAAGAAGGCAGCAG |
|  |  | GCCTGGTCATTGGCATACTT |
| PpUFGT  (ppa005162m) | 121 | CCGCTGCCTCTCCCAACACTC |
|  |  | CCATCAGCCACATCAAACACCTTTAT |
| NtEF2  (ppa001368m) | 129 | GGTGTGACGATGAAGAGTGATG |
|  |  | TGAAGGAGAGGGAAGGTGAAAG |
| NtFLS  (AB289451) | 172 | AAGGATGGCCATTGGTATGA |
|  |  | CTGATGGTGGCTCCAAGAAT |
| NtLAR  (AM827419) | 190 | ACGAACCTGCTTCTCTTTGG |
|  |  | TCAAGGTCCTTTACGCCATC |
| NtANR  (AM791704) | 107 | CATGTGGAAGATGTTTGTCG |
|  |  | TTTGCTAGCTCCGGAACACT |
| NtUFGT  (GQ395697) | 124 | CAATGTTTGGGATGGTGTCA |
|  |  | TTCCTCCTCTGCCTCTTTCA |
| NtActin  (X69885) | 153 | CTGAGGTCCTTTTCCAACCA |
|  |  | TACCCGGGAACATGGTAGAG |
| *[Vector contruction]* | | |
| PpMYB10.1 ORF | 720 | ATGGAGGGCTATAACTTGGGTGT |
|  |  | TTAATGATTCCAAAAGTCCACGTTAA |
| PpMYB10.2 ORF | 684 | ATGGAGGGTTATGACTTGAGT |
|  |  | TTACTTTCTATATTCTTCATTTGAATGA |
| PpMYB10.3 ORF | 738 | ATGGGGGGAAATAACTTGGA |
|  |  | TTATTCTTCTTTTGAATGATTCCAAAGGTC |
| PpbHLH3 ORF  (ppa002884m) | 1875 | ATGGCTGCACCGCCAAGTAG |
|  |  | CTAGGAATCAGATTGGGGAATTATTTGA |
| PpANS promoter | 2000 | TCATATATAGTCACATCAACCGTCTTTAC |
|  |  | TTTGGCAGCCGGCTCTTCGTAA |
| PpUFGT promoter | 2000 | TACAACCTGAAATTCTTCCTTGGCTCT |
|  |  | ATATGTATGAGCTAATAAGACTAATTGGAG |
| *[MYB10.1 type discrimination]* | | |
| MYB10.1 alleles | P1-P2: 609  P2-P3: 426 | P1: GGGAAACGATGTAAAGCCAC |
|  |  | P2: CGAATATCAATGCAGCATCGTG |
|  |  | P3: CGGTTTTGGTCTTGCGCTAT |
| MYB10.1 transcripts | 568 | GAAGAGCTGTAGACTAAGGTG |
|  |  | GTCCACGTTAAAAGAGAAATCAC |
